# ‘Exploring the Influence of Social Media Influencers on Intention to Attend Cervical Screening in the UK: Utilising the Theory of Planned Behaviour’

**DOI:** 10.1177/10732748221079480

**Published:** 2022-04-09

**Authors:** Naomi Fielden, Patricia Holch

**Affiliations:** 1 Department of Psychology, 4467 Leeds Beckett University, Leeds, West Yorkshire, UK

**Keywords:** cervical screening, influencer, health promotion, health protective behaviour, social media, theory of planned behaviour, Jade Goody effect

## Abstract

**Objectives:**

Cervical cancer is 99.8% preventable when detected early; however, uptake of screening in the United Kingdom is at a 20-year low. Recently, a number of social media influencers have video logged about their experiences of cervical screening through narrative communication with their audience. Here we aimed to explore if accessing cervical screening information from a social media influencer can impact the theory of planned behaviour variables and predict intention to attend cervical screening appointments.

**Design:**

Utilising a cross-sectional design a volunteer sample of 102 UK women (mean age = 28; SD = 3.10; range = 25–35) took part in an online questionnaire study.

**Results:**

Hierarchical regression modelling revealed attitude as a significant predictor of intention to attend a cervical screening appointment and that social media influencers affect attitudes of their audience, indirectly influencing intention to attend.

**Conclusion:**

Health messages communicated by social media influencers are effective in promoting positive attitudes but not directly influence intention to attend towards cervical screening. Further research should explore influencer impact on attitudes towards this health behaviour with the ultimate aim of increasing attendance and consequently saving lives.

## Introduction

Cervical cancer is the second most prevalent cancer among women worldwide^1^ and is 99.8% preventable.^2^ In the xx, 3200 reported incidences per year result in 850 deaths.^3,4^ Incidence increases after age 25^5^ with the highest in those aged 30–34 and 25–29.^3^Cervical screening is vital to detection and saving lives. Women in the xx over 24 are invited every 3 years, and in 2019–2020, 4.63 million were eligible.^6^ However, the xxxxxx (xxx) in the x has seen differing patterns of screening attendance.^7^

Half a million additional cervical screening attendances occurred between 2008 and 2009 during the media reporting of the xx celebrity, Jade Goody, who died from the disease aged 27 (‘The Jade Goody effect’).^8^ However, current attendance is at a 20-year low,^9^ particularly in the most at risk 25 to 35 age group; equating to almost a quarter of a million young women.^4,10^ With the Jade Goody effect no longer current^11^ and the low uptake of screening, it is important to explore how health promotion can be targeted to reach them.

Cervical screening was traditionally promoted via leaflets and TV campaigns,^12^ with the general practitioner (GP) pivotal.^13^ Now, patients are actively encouraged to manage their own health^14^ and seek information online.^15-17^ Indeed, there is some evidence that targeted social media interventions can improve cancer awareness, screening intentions and uptake.^18^

This change has coincided with a rise in celebrity internet ‘influencers’ promoting products and lifestyle change.^19,20^ Influencers, however, can have a prominent effect on health promotion and behaviour.^21,22^ Marlow et al.^23^ argue that influencers offer a style of ‘narrative communication’ enabling a memorable social connection. (previously seen with the ‘Jade Goody effect’). Previously, Kreuter et al.^24^ found narrative communications (testimonials and storytelling) were important tools for cancer prevention and control. This type of communication was evident when fashion blogger Sarah Ashcroft, and YouTube star Zoë Sugg, video logged (a blog with mostly video content) preparing for, attending and sharing feelings and fears whilst having their cervical screening appointments.^25,26^ This type of content creates an intimate relationship with influencer and audience^27^ and is pivotal to influencer success in changing audience behaviours.^28^

However, not all influencer health advice is credible, and they may be sponsored to produce content (SponCon); indeed, influencers were paid to advocate Allergan breast implants when they had been withdrawn from circulation by the Federal Drugs Agency (FDA).^29^ The potential access to untrustworthy information is a cause for concern, particularly as young adults are often not health literate.^30^ However, being able to predict individual’s intention to perform healthy behaviours is key.

The theory of planned behaviour (TPB) is the most widely used model for predicting intention^31-35^ and as a precursor of behaviour.^36^ The TPB has wide applicability.^37-40^ In the TPB attitudes, subjective norms and perceived behavioural control (PBC) are key. In terms of screening behaviours, women are more likely to intend to attend screening if they have a positive attitude to it^41,42^ and if they believe that significant others will approve (subjective norms).^43^ If they feel they have good access to screening (perceived behavioural control), they are more likely to attend.^14^ Anticipated regret (regret they may feel in the future) has been recently added^42,44-46^ and has strengthened the predictive power for intentions^47,48^ and the intention-behaviour relationship.^49^

Social media influencers have the capacity to influence the TPB constructs to shape audiences intentions^19,50,51^ as they are now viewed as part of individual’s social networks, influencing both social^52^ and health related norms.^53^ Influencers also impact PBC by demonstrating the ease of attending a cervical screening appointment^26^ breaking down perceived barriers.^42^ As more individuals turn to the internet for health advice, we explore the impact social media influencers have on the subjective norms, attitude, PBC, anticipated regret and intention to attend a cervical screening appointment in xx women over 25.

## Method

### Design

A cross-sectional design was employed utilising linear regression modelling. The outcome variables were the intention to attend cervical screening and the 4 predictor variables were as follows: subjective norms, attitude, PBC, anticipated regret (continuous) and previous exposure to ‘social influencers’ on social media speaking about the subject of cervical screening (SMIE) (a dichotomous variable). Participants were dichotomised into groups depending on whether they answered ‘yes’ or ‘no’ to the question ‘*have you viewed a social media influencer talking about cervical screening’* (SMIE) and labelled the ‘*exposure to an influencer group’* (n = 62) and the ‘*no exposure to an influencer group’ (n = 40)*. For the remaining analysis, the participants were analysed as a whole group.

### Recruitment and Sample Size

Participants were recruited as a volunteer sample where they responded to the study details (summary about the project and a screenshot of the recruitment poster) through extensive sharing via social media (Facebook, Twitter and Instagram), further an email and recruitment poster was shared though the organisation Mercy xx and around the Leeds Beckett University (LBU) campus.

To ensure sufficient statistical power, the following sample size calculation was undertaken outlined by Cohen^54^ for multiple regression with power set at .80 and an α = .05. Thus, to gain a medium effect size with 5 predictors, a total sample size of 91 was required.^55^

### Measures

Participants completed a demographics questionnaire and the TPB questionnaire based on cervical screening behaviours by Walsh et al.^42^ (see supplementary information) adapted to include questions about social media influencers. This questionnaire was validated by a sample of women in Ireland (N = 3000) with Cronbach’s α values above .5, 42. The TPB/TRA was originally developed by Fishbein and Ajzen^34,56^ and has been used widely in health-related research including research into cervical screening.^57-60^

Questions assessing attitudes, subjective norms, PBC and anticipated regret were measured on 5-point scales. Attitude was measured by responses to the question: ‘*For me, going for a cervical screening appointment within the next 3 months would be…’* using 8 adjective scales *(reassuring, unpleasant, embarrassing, unwise, important, worrying, worthwhile and healthy)*. Subjective norms by the responses to ‘*most people who are important to me would think that I should go for a cervical screening appointment within the next 3 months’* and ‘*most people who are important to me would approve of me attending for a cervical screen in the next 3 months if I am given the chance’.* PBC the responses to: ‘*How easy or difficult would it be for you to go for a cervical screening appointment within the next 3 months?’* The second question being ‘*How confident are you that you will be able to go for a cervical screening appointment within the next 3 months’*. Finally, intention was measured by the responses to: ‘*I intend to go for a cervical screening appointment within the next 3 months’* and ‘*I will try to go for a screening appointment within the next 3 months’.* Anticipated regret was measured using 5 items using the question: ‘*How would you feel if you did not attend for a smear test in the next 3 months when given a chance?’* on 5 items (*anxious, tense, guilty, worried and regretful*)*.*

The questions were scored from 1 to 5, and reverse scored if they were a positive statement. Therefore, the higher the participant’s score, the more favourable the social norms, PBC, attitude and intention to attend for a cervical screening appointment. The grouping of participants and then dichotomous variable of whether the participant had viewed an influencer talk about cervical screening was coded as 0 = no and 1 = yes.

### Procedure

The study was conducted online, and the questionnaires (demographics and TPB questionnaire) were administered via the online via the online questionnaire builder Qualtrics™. Prior to the TPB questionnaire, participants were asked about whether they had viewed an influencer talk about cervical screening. Post questionnaire completion, participants were debriefed and thanked for their time. The anonymised data were downloaded from Qualtrics™ directly into IBM SPSS version 26.

### Ethical Considerations

Ethical approval from Leeds Beckett University (LBU) was obtained on 16.01.2020 (67 875). The study conformed to the Association of Internet Researcher’s and British Psychological Society’s ethical guidelines on Internet Mediated Research (IMR).^61^

### Missing Data

Outliers were removed and 55 participants were excluded due to ≥40% incomplete responses. The remaining missing values accounted for 1.7% of the final data set, and these were not imputed due to the potential impact on reliability and validity.^62^

### Statistical Analysis

The data were exported from Qualtrics into SPSS (V. 26) and relevant assumption checks performed. A one-way ANOVA was conducted with exposure to an influencer and no exposure to an influencer as factors. Following correlation analyses on predictor (attitudes, subjective norms, perceived behavioural control and anticipated regret and previous exposure to ‘social influencers’ on social media speaking about the subject of cervical screening) and outcome variable (intention to attend cervical screening), a hierarchical multiple regression was performed. We anticipated the TPB variables to covary.

## Results

### Participants

One hundred and two female xx residents aged between 25 and 35 (mean age = 28 years; SD = 3.10) (see Table 1) were recruited were recruited as these are the target population with low screening uptake.^4,10^

**Table 1.** *Sociodemographic characteristics of the sample dichotomised by exposure to social media (*N *= 102)*.

Demographic	Exposure to an influencer (N = 62)	No exposure to an influencer (N = 40)	Total
**Mean age in years (SD)**	27 (3.33)	29 (2.75)	28 (3.10)
**Marital status mean (%)**			
Single	28 (45.16%)	21 (52.5%)	49 (48.04%)
Married	15 (9.3%)	14 (35%)	29 (28.43%)
Living with partner	19 (30.65%)	4 (10%)	23 (22.5%)
Divorced	1 (1.61%)	0 (0%)	1 (.98%)
**Education mean (%)**			
Degree/higher degree	37 (59.68%)	26 (65%)	63 (61.76%)
A level, highers/equivalent	19 (30.65)	7 (17.5%)	26 (25.49%)
BTEC/GNVQ	3 (4.84%)	4 (10%)	7 (6.86%)
GCSE (Grade A–C)	2 (3.23%	2 (5%)	4 (3.92%)
GCSE (Grade D–G)	1 (1.61%)	1 (2.5%)	2 (1.96%)
**Employment mean (%)**			
Employed (full-time)	29 (46.77%)	23 (57.5%)	52 (50.98%)
Employed (part-time)	13 (20.97%)	5 (12.5%)	18 (17.65%)
Self-employed	5 (8.06%)	0 (0%)	5 (4.9%)
Unemployed	2 (3.23%)	6 (15%)	8 (7.84%)
University student	12 (19.35%)	5 (12.5%)	17 (16.67%)
College student	1 (1.61%)	1 (2.5%)	2 (1.96%)
**Ethnicity mean (%)**			
White British	59 (95.16%)	36 (90%)	95 (93.14%)
Other white background	1 (1.61%)	1 (2.5%)	2 (1.96%)
White and Asian	1 (1.61%)	0 (0%)	1 (.98%
Black African	0 (0%)	2 (5%)	2 (1.96%)
Mixed background	1 (1.61%)	0 (0%)	1 (.98%)
Other	0 (0%)	1 (2.5%)	1 (.98%)

Equal variances were identified across the sample for intention, PBC, anticipated regret and subjective norms (P ≥ .54); however, unequal variances were identified in attitude (P = .046). Therefore, for attitude, a Spearman’s *Rho* correlation coefficient was utilised. Internal consistency scores for subjective norms were acceptable (α = .61), for attitude and PBC good, (α = .76; α = .70) and for intention moderately low (α = .56). ANOVA is relatively robust to violation of this assumption when sample sizes are relatively equal and no group exceeds a ratio of 4:1 for largest to smallest^63^ as in this current study. The Skewness and Kurtosis values showed that for all variables, skewness (≥−1.28) and kurtosis (≥−1.84) values are between +2 and −2 demonstrating a normal distribution.^64^

An analysis of variance (ANOVA) was performed and there was no significant effects at P < .05 for intention (F (1,97) = .65, P = .423), PBC, (F (1, 100) = 1.15, P = .287), subjective norms (F (1,100) = 2.2, P = .141) or anticipated regret (F (1, 69) = 1.49, P = .226). However, a significant effect was demonstrated for exposure to influencers for attitude (F (1, 95) = 4.42, P = .038), with the mean score for the exposure group (M = 25.6, SD = 1.83) being significantly higher than the no exposure group (M = 24.6, SD = 2.37), (See Table 2)

**Table 2.** *Means (M) and standard deviations (SD) of the major study variables and ANOVA (*N*=102)*.

Variable	Exposure to an influencer (N = 62)	No exposure to an influencer (N = 40)	ANOVA *P* value
	Mean (SD)	Mean (SD)	
Intention	7.25 (2.06)	6.87 (2.6)	423
Attitude	25.55 (1.83)	24.65 (2.37)	045*
PCB	8.94 (1.52)	8.58 (1.85)	941
SN	7.95 (1.38)	7.53 (1.47)	.141
AR	14.23 (5.41)	12.48 (6.51)	.226

*significant at <.05.

SD = standard deviation. PBC = perceived behavioural control; SN = subjective norm; AR = anticipated regret.

Table 3 presents the Pearson and Spearman correlation coefficients for the TPB variables. Social media influencer exposure was included as a dichotomous variable and performed as a point-biserial correlation. Intention was significantly correlated with attitude, perceived behavioural control and anticipated regret (P < .001) however not to social norms nor exposure to a social media influencer. Social norms and perceived behavioural control were significantly correlated (P < .001) but not to any other variable. Table 4 presents the percentage of the total participant sample who had gained information about cervical screening from each of the sources below, and participants could select more than 1 choice. It was clear that primary care was the most popular source for cervical screening information with 60.8% of the sample sourcing information for GPs and 45.1% from practice nurses; however, 42.2% said they gained information from the internet, family and friends were (28.4 and 24.5%, respectively) and newspapers only 6.9%. A chi-square test of independence with Fisher’s exact test a significant association between viewing a social media influencer and gaining information from a doctor χ2 (1, n = 102) = .3.8, P = .04 and from friends χ2 (1, n = 102) = .5.8, P = .013. For the other categories, no significant associations were found χ2 (1, n = 102) ≤ 2.51, P ≤ .272.

**Table 3.** *Spearman’s correlations of the major study variables (*N *= 102)*.

Variable	1	2	3	4	5	6
1.Intention	-	.33**	.27**	.07	−.08	.33**
2.Attitude	.33**	-	.35**	0	−.18	.36**
3.PBC	.27**	.35**	-	.45**	−.11	.31**
4.SN	.07	0	.45**	-	−.15	.1
5.SMIE	−.08	−.18	−.11	-.15	-	−.15
6. AR	.33**	.36**	.31**	.1	−.15	-

**Correlation is significant at the .01 level (2-tailed).

PBC = perceived behavioural control; SN = subjective norms; SMIE = social media influencer exposure; AR = anticipated regret.

**Table 4.** *Participants sources of information regarding cervical screening (*N *= 102)*.

Source of information about cervical screening	Percentage of participants, %
Doctor	60.8
Practice nurse	45.1
Internet	42.2
Family	28.4
Friend	24.5
Newspaper	6.9
Television	0
Radio	0

All assumptions prior to proceeding to regression modelling were met including Cook’s distance, lack of multicollinearity, independence of errors, lack of homoscedasticity, normally distributed error and non-zero variances.

### Hierarchical regression

#### Regression

Table 5 shows the hierarchical regression scores for each predictor: attitude, social norms, PBC and anticipated regret. Attitude was initially entered and this model was statistically significant F (1, 68) = 6.25; P = .015 explaining 8.4% of the variance of intention, followed by subjective norms and PBC and this model was not statistically significant F (3, 66) = 2.03; P = .118 and did not contribute to the variance of intention. Anticipated regret was added and was not statistically significant F (4, 65) = 2.39; P = .060 but contributed to 4.3% of the variance.

**Table 5.** *Hierarchical regression scores (*N *= 102)*.

Predictor	β	*B*	*t*	*SE*	*R*	*R* ^*2*^	*R* ^*2*^ *change*
**Step 1**					.29	.08*	
Attitude	.29*	.31	2.5	.12			
**Step 2**					.29	.09	.00
Attitude	.28*	.3	2.16	.14			
SN	.02	.02	.12	.19			
PBC	.01	0.1	.05	.18			
**Step 3**					.36	.13	.04
Attitude	.23	.24	1.68	.14			
SN	.03	0.3	.18	.19			
PBC	−.04	−.04	−.23	.18			
AR	.23	.08	1.8	.04			

Note. Statistical significance: *P < .05; SMIE = social media influencer exposure; PBC = perceived behavioural control; SN = subjective norm; AR = anticipated regret; dependent variable = intention.

## Discussion

Here we predicted there would be a significant difference in the attitude, PBC, subjective norms and anticipated regret of the participants who had been exposed to a social media influencer talking about cervical screening information than those who had not. We also predicted that the TPB variables and being exposed to a social media influencer talking about cervical screening information would each have a significant predictive effect on the intention to attend a cervical screening appointment. There was partial support for this, in that attitude was found to be the sole significant predictor of intention to attend cervical screening but explaining only 8.4% of the variance. Social norms, PBC, anticipated regret and being exposed to an influencer were not significant predictors of intention. Similarly, only attitudes were found to be significantly higher (more positive) in the exposure group compared to the no exposure group.

However, viewing an influencer talk about cervical screening was not a significant predictor of intention to attend an appointment, at odds with previous literature regarding social media influencers’ effect on intentions.^51,65-67^ Although, this research often surrounded ‘purchase’ intention as opposed to the intention to participate in health behaviours. Similarly, research within the health behaviour domain which demonstrated effects of social media exposure on intentions largely focused on diet and exercise rather than behaviours involving medical contact.^68^ This may imply that influencers can only affect intentions for medical self-care and purchasing a product rather than attending a cervical screening appointment where barriers to attendance include fear of the findings and fear of pain.^69^ To support this assertion, most women in this study would still access their doctor for cervical screening information (60.4% of participants), followed by a practice nurse (45.1%), then the internet (42.2%). Indeed our findings also show that those who had viewed a social media influencer also were significantly more likely to speak to a GP. Thus, traditional sources are still valued for young women and they have not migrated away as previous research has claimed.^16,68^ This is supported by a recent survey that 9 out of ten individuals still have ‘confidence and trust’ their GPs.^70^

However, attitude scores towards cervical screening were found to be significantly higher (and thus more positive) in the exposure to an influencer group and attitudes were also a significant predictor of intention. This supports previous research that influencers have the ability to shape the attitudes of their audience.^19,71^ Thus, viewing an influencer video log or talk about screening information may have an indirect rather than a direct influence on cervical screening. This is an interesting finding as it does reveal a link with a potential influence on health protective behaviours.

In terms of the TPB, these findings support previous research from Marteau et al.^72^ and Godin and Kok^73^ who found attitude to be a strong predictor of cervical screening intention and health protective behaviours^74^ and that attitudes had a greater influence over intentions than the other TPB constructs^75^ conforming they are fundamental to behaviour.^76^ Thus, changing attitudes may be the most effective way to increase attendance for this particular age group.

In contrast, PBC and subjective norm scores were not found to be significantly different in between the 2 groups. This was surprising as previous research found that both variables could be impacted by influencers.^28,52^ This implies that the content provided about cervical screening by influencers is not affecting these constructs. Considering PBC with reference to the TPB, this current study did not find this to be a significant predictor of cervical screening attendance. A finding partially supported by the previous meta-analysis from Cooke and French^75^; however, Godin and Kok^73^ found intentions were strongly associated with PBC when applied to screening attendance; however, it must be noted the review is not current. Thus, the previously identified barriers preventing cervical screening attendance such as ease if information access and costs (due to time away from work or travel)^42^ may not be relevant now. In the xx, cervical screening is free and accessible via internet booking and offered by doctors, nurses and within specialist clinics. For subjective norms, the current findings are contrary to previous research highlighting that this was strongest predictor of intention and behaviour in cervical screening uptake.^75,77^ Targeting social norms has been the focus of xxx campaigns to increase cervical screening attendance, but as demonstrated here, this would not be an effective intervention for this age group

Furthermore, anticipated regret was also not found to be significantly different between the exposure to an influencer group and the no exposure group. No previous research had explored the direct relationship between influencers and anticipated regret, and this was inferred from previous research into exemplar regret and observers being more likely to partake in the behaviour they observed the individual not doing.^78^ Alternatively, the material being presented by influencers may not highlight anticipated regret, talking mainly about their experience of attending rather than the consequences of not attending. Additionally, we also found that anticipated regret was not a significant predictor of the intention to attend cervical screening. This opposes the results of previous studies including Walsh et al.^42^ where ‘anticipated regret’ significantly added to the model^46,47,79^ and that intention to seek medical help for cancer is associated with higher levels of anticipated regret.^80^

Thus, the TPB model is not completely supported by the current study as this model suggests all 3 factors: subjective norms, PBC and attitude to be all significant predictors of intentions. Additionally, the variables only accounted for 14% of the variance in intention to attend a cervical screening appointment, much smaller than the 41% found in the Walsh et al.’s^42^ study. This suggests for the current study’s demographic, and it highlights there may be other factors that are also significant predictors (not yet identified) of intention for this age group to perform this particular health behaviour. Future studies should explore whether screening behaviour has actually been enacted and explore the motivation for attending to enable further exploration of a potential behaviour-intention gap.

A limitation of this study is that it did not explore whether the behaviour was actually performed, focussing only on intention. Intentions do not always lead directly to performing a behaviour; the behaviour-intention gap defined by Sheeran and Webb^81^ suggested approximately only one half of intentions translate into behaviour. It is possible that social desirability could have affected the results; however, we aimed to minimise this by using an online survey with validated instruments and reassuring participants that the data was anonymised and using validated instruments. Further, the study only focussed on a small demographic of predominantly white xx women, this does not reflect the social norms of the diverse xx population.^82^ A further limitation is the cross-sectional design, which provides only a snapshot of behaviours and intentions future research should employ longitudinal designs to explore behaviours and actual intentions over time.

However, the current study does explore an area which has not yet been clearly investigated and provides insight into the potential impact social media influencers can have on serious health decisions such as cervical screening. These findings suggest impact differs dependent on the level of importance. For example, influencers may successfully impact health protective behaviours such as dieting and exercise; indeed, there is a great deal of non-professional health updates accessed on social media particularly around the healthy diet discourse^17^ but their views may not be as trusted when it comes to medical checks such as cervical screening. However, they may indirectly influence intentions through shaping the attitudes of their audience. It would be pertinent to explore the mechanisms of how ‘influencers’ influence in relation to cervical screening intentions and if the ‘similarity-attraction effect’^83^ is a factor, where individuals are more likely to take the advice of someone who was culturally similar to them. Knowledge of the extent to which influencers can impact different types of health behaviours would help inform effective health promotion campaigns. However, whilst there could be a role for influencers with regard to health messaging, it is important that any campaign involving them in the future should be robustly linked to the xxx and be free from sponsorship. It is also important to identify which platforms women target users are currently more likely to engage with when designing social media interventions.^18^ This is particularly true for harder to reach ethnic minorities or disabled women a currently priority for Jo’s Trust in the xx.^84^

## Supplementary Material

Supplementary material

## Conclusion

Women aged 25 to 35 in the xx currently have the highest cervical cancer incidence rate and the lowest levels of screening attendance. The finding that attitude is a significant predictor of intention to attend a cervical screening appointment and that influencers were able to indirectly impact intentions (by influencing attitude) suggest future health messaging should target attitudes about cervical screening with this age group. This could be communicated by social media influencers within xxx campaigns as an indirect but effective way of forming more positive attitudes to cervical screening, with the ultimate aim of increasing attendance and saving lives.
