# Supplementary material for: ‘Exploring the Influence of Social Media Influencers on Intention to Attend Cervical Screening in the UK: Utilising the Theory of Planned Behaviour’
Source: Cancer Control. 2022 Apr 9;29:10732748221079480. doi: 10.1177/10732748221079480 (PMC8998370; doi:10.1177/10732748221079480)
Supplement: Supplementary material [file Cervical.docx]

**CERVICAL SCREENING QUESTIONNAIRE**

**1.    Have you ever had a cervical screening appointment**

**Yes                No   Don’t know**

**2.    If not, why not?**

**3.    If yes, when did you have your last cervical screening appointment? (Please tick one of the following)**

**Within past 12 months**

**Within past 3 years**

**3-5 years ago**

**5-10 years ago**

**Over 10 years ago**

**Do not know**

**4.    Approximately how many screens do you think you have had in your lifetime to date?**

**5.    What do you believe are the main purposes of a cervical screen?**

**(please tick as many as you believe are correct)**

**To detect infections**

**To detect cervical cancer**

**To prevent cervical cancer**

**To detect sexually transmitted diseases**

**To detect changes in the cells of the cervix**

**Don’t know**

**Other (please state)_______________________**

**6.     In the past, where did you get your information about a cervical screening?**

**Doctor                              Practice Nurse**

**Family                            Friends                                     Radio                                T.V.**

**Newspaper                     Internet**

**7.    Do you feel that you have had sufficient information on cervical screening in the past?**

**Yes                    No**

**8. Have you ever viewed a social media influencer talk about cervical screening?**

**Yes                   No**

**9. If so, which platform was it presented on?**

**Instagram**

**Facebook**

**Twitter**

**Pinterest**

**Snapchat**

**Other**

**10.    Do you have a family history of cervical cancer?**

**Yes                  No**

**PLEASE ANSWER THE FOLLOWING QUESTIONS BY CIRCLING THE ANSWER OF YOUR CHOICE**

**11.    It is unlikely that I will get cervical cancer, even if I do not go for a cervical screen**

**Strongly agree                      Agree Unsure         Disagree Strongly disagree**

**12.  I do not think that I am personally at risk of cervical cancer**

**Strongly agree                      Agree Unsure           Disagree Strongly disagree**

**13.  I am afraid to have a cervical screen**

**Strongly agree                       Agree Unsure         Disagree Strongly disagree**

**14.  Having a cervical screening test is too inconvenient for me**

**Strongly agree                    Agree Unsure         Disagree Strongly disagree**

**15.  Getting a cervical screening test does not interfere with my other activities**

**Strongly agree                      Agree Unsure         Disagree Strongly disagree**

**16.  Getting a smear test is time consuming**

**Strongly agree                      Agree Unsure           Disagree Strongly disagree**

**17.  The way a cervical screen is performed causes me distress**

**Strongly agree                     Agree Unsure           Disagree Strongly disagree**

**18.  I feel at ease when having a cervical screen**

**Strongly agree                    Agree Unsure         Disagree Strongly disagree**

**19.  I am too old to have a cervical screen**

**Strongly agree                  Agree Unsure        Disagree Strongly disagree**

***20.* Most people who are important to me would think I should attend for a cervical screen in the next 3 months if I am given the chance**

**Strongly agree               Agree Unsure          Disagree Strongly disagree**

***21.* Most people who are important to me would approve of me attending for a cervical screen in the next 3 months if I am given the chance.**

**Strongly agree             Agree Unsure       Disagree Strongly disagree**

**22.  What has your experience of cervical screening to date been like?**

**…..good      *extremely              very         fairly slightly       not at all***

**…..reassuring   ex*tremely       very         fairly slightly      not at all***

**…..unpleasant    e*xtremely    very        fairly slightly     not at all***

**…..embarrassing *extremely  very     fairly slightly     not at all***

**…..uncomfortable  *extremely  very   fairly slightly   not at all***

**…..painful   *extremely    very         fairly slightly   not at all***

***23.*  Attending for a cervical screening appointment in the next three months if given the chance would be….**

**…..worrying   *extremely  very         fairly slightly     not at all***

**…..reassuring    *extremely  very     fairly slightly      not at all***

**…..unpleasant  *extremely  very   fairly slightly     not at all***

**…..healthy  *extremely      very   fairly slightly     not at all***

**…..embarrassing   *extremely  very   fairly slightly   not at all***

**…..unwise   *extremely    very         fairly slightly not at all***

**…..important   *extremely   very         fairly slightly    not at all***

**…..worthwhile   *extremely   very     fairly slightly    not at all***

***24.* How easy or difficult would it be for you to attend for a cervical screen in the next 3 months if given the chance?**

***Very easy       Fairly easy Unsure     Fairly difficult Very difficult***

***25.* If you were given the chance, how confident are you that you could attend for a smear test in the next 3 months?**

***Very confident     Fairly confident     Unsure     Fairly unconfident Very unconfident***

***26.* If I wanted to, I could easily go for a cervical screening appointment within the next 3 months**

***Strongly agree                       Agree Unsure                   Disagree Strongly disagree***

***27.* How would you feel if you did not attend for a cervical screening appointment in the next 3 months when given the chance?**

***Extremely anxious              Very anxious       Unsure Slightly anxious   Not at all anxious***

***Extremely tense Very tense         Unsure Slightly tense       Not at all tense***

***Extremely guilty                 Very guilty         Unsure Slightly guilty        Not at all guilty***

***Extremely worried             Very worried       Unsure Slightly worried    Not at all worried***

***Extremely regretful           Very regretful       Unsure Slightly regretful  Not at all regretful***

**28.  How likely is it that you will attend for a cervical screen in the next 3 months if given the chance?**

***Extremely Likely Very likely     Unsure Fairly unlikely        Extremely unlikely***

**29.  I intend to go for a cervical screen within the next 3 months.**

***Strongly agree       Agree Unsure        Disagree Strongly disagree***

**30.  Would any of the following prevent you from going for a cervical screening appointment within the next 3 months? (Please tick those that apply to you)**

**o  Lack of time**

**o  Other commitments, such as work or family to look after**

**o  Difficulty getting to surgery, such as lack of transport or health problems**

**o    Unsuitable appointment times**

**o    Male smear taker**

**31.  You are more likely to go for a cervical screen if you decide where and when you will go.  Please write in below when, where and how you will make and appointment to go for a screen.**

**When I will make the appointment**

**Date…………………………………..**

**Time………………………………….**

**How I will make an appointment e.g. by phone, in person**

***Please write in here………………………………………………***

**Where the appointment will be made for, e.g. name of practice/surgery.**

***Please write in here……………………………………………***

**THANK YOU FOR YOUR CO-OPERATION IN FILLING IN THIS QUESTIONNAIRE**
